# Outcomes of Intranodal and Modified Intranodal Lymphangiography for Treatment of Traumatic Chylous Leaks in the Thorax and Neck

**DOI:** 10.1007/s00270-024-03900-z

**Published:** 2024-12-10

**Authors:** Rupal Parikh, Elisabeth R. Seyferth, Sanjay Palat, Maxim Itkin, Gregory J. Nadolski

**Affiliations:** 1 Division of Interventional Radiology, Department of Radiology, University of California San Diego School of Medicine, San Diego, CA 92037 USA; 2 Division of Interventional Radiology, Department of Radiology, Perelman School of Medicine at the University of Pennsylvania, Philadelphia, PA USA; 3 Department of Medicine, Washington University School of Medicine, St. Louis, MO USA

**Keywords:** Lymphangiography, Thoracic duct embolization, TDE, Intra-nodal lymphangiography, Chylous leak, Chylothorax

## Abstract

**Purpose:**

To report outcomes, procedure and fluoroscopy times, and adverse event rates after intranodal lymphangiography (IL) and modified IL (mIL) for treatment of traumatic chylous leaks in the thorax and neck.

**Methods:**

Under an IRB-approved protocol, retrospective review of a quality assurance database identified all lymphangiograms for post-surgical refractory chylous leaks in the thorax and neck at a tertiary center from 2002–2022. Records were reviewed for technical and clinical outcomes, procedure and fluoroscopy times, and adverse events. Pedal lymphangiograms were excluded. Patients were categorized into IL (pre-2016) and mIL (post-2016) cohorts. mIL incorporated pneumatic calf compression throughout the procedure. Technical success was defined as successful thoracic duct cannulation. Clinical success was defined as leak resolution and eventual chest or other drain removal within 2 weeks post-procedure. A two-tailed Fischer’s exact test was used to compare categorical outcomes. A two-tailed *t* test was used to compare means.

**Results:**

Two hundred and thirty-nine patients underwent 263 thoracic duct embolizations of traumatic chylous leaks in the thorax/neck. Intranodal lymphangiography was used in 167 cases in 150 patients. Overall clinical success was 94.6% [n = 142/150]. Technical success was higher in mIL (94.2% [81/86]) than IL (76.5% [62/81]) (*p* = 0.002). Clinical success per patient and procedure were similar between cohorts (92.3% [72/78] mIL versus 97.2% [70/72] IL, *p* = 0.27, and 83.7% [72/86] mIL versus 85.1% [69/81] IL, *p* = 0.83, respectively). Mean procedure time in mIL (83.4 ± 31.9 min) was shorter than in IL (119.2 ± 45.9 min) (*p* < 0.0001). Mean fluoroscopy time in mIL (33.8 ± 17.3 min) was shorter than in IL (41.7 ± 23.2 min) (*p* = 0.02). Adverse event rate was not significantly different between groups.

**Conclusion:**

Overall, thoracic duct embolization for traumatic chylothorax has high clinical success, approaching 95%. While clinical success of mIL was similar to IL, technical success and mean procedure and fluoroscopic times were significantly improved. Findings suggest modified intranodal lymphangiography should be utilized to treat traumatic chylothorax.

**Level of Evidence:**

Level 4, Case Series.

**Graphical Abstract:**

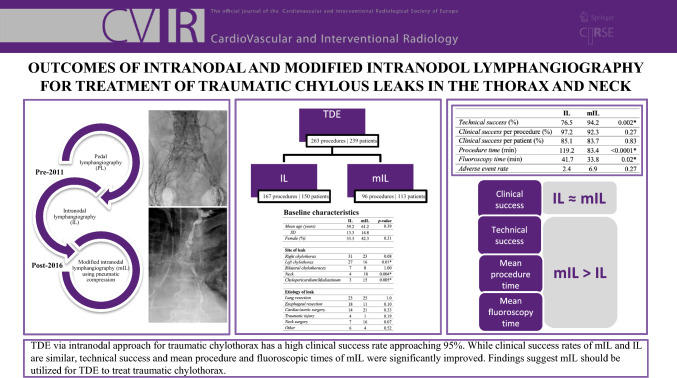

## Introduction

Thoracic duct embolization (TDE) is a percutaneous, minimally invasive treatment alternative to surgical ligation in patients with post-operative chylous leaks refractory to conservative management [1]. In the past, thoracic duct (TD) opacification for identification and embolization of a lymphatic leak was achieved with the use of pedal lymphangiography (PL) [2]. PL was a technically demanding approach requiring isolation and cannulation of pedal lymphatics to administer lipiodol into the extremities and abdomen/pelvis prior to TD opacification and cannulation [3].

In contrast, intranodal lymphangiography (IL) involves ultrasound-guided access of the bilateral inguinal lymph nodes and administration of ethiodol at the inguinal level, bypassing the lower extremities and obviating the need to isolate and cannulate the pedal lymphatics [4]. Furthermore, a modified IL-approach with application of pneumatic calf compression devices has been described [5]. While this initial description of mIL reported decreased ethiodol transit times suggesting improved efficiency, it included some subjects without lymphatic leaks undergoing lymphangiography as well as non-traumatic chylous leaks. Thus, technical and clinical success of mIL in the setting of TDE compared to unmodified IL technique for traumatic chylothorax remains incompletely reported.

The present study reports the procedure time, fluoroscopic time, technical outcomes, and clinical outcomes for patients undergoing TDE via an intranodal approach for refractory post-surgical chylous leaks in the thorax or neck, comparing outcomes between IL and mIL approaches to TDE.

## Materials and Methods

### Patients

This study was approved by the institutional review board and is compliant with standards set forth by the Health Insurance Portability and Accountability Act. A retrospective review of a quality assurance database (Hi-IQ: ConexSys, Lincoln, RI) identifying all patients referred for TDE to treat refractory post-surgical chylous leaks in the thorax or neck at a single tertiary care center between 2002 and 2022 was performed. Patients who underwent a pedal approach to lymphangiography were excluded. All subjects who underwent intranodal lymphangiography for attempted TDE were included. All patients had chylothorax diagnosed by presence of white fluid from a chest tube or surgical drain with triglycerides > 150 mg/dL. Patients were categorized into IL (pre-2016) and modified IL (mIL, post-2016) cohorts. mIL incorporated the addition of pneumatic compression devices on the calves during and throughout lymphangiography and TDE. Baseline patient characteristics, including etiology and location of leak, are detailed in Table 1. The groups are not significantly different in age (*p* = 0.39), sex (*p* = 0.31), or etiology of chylous leak.

**Table 1:** Baseline characteristics

	IL	mIL	*p value*
*Mean age (years)*	59.2	61.2	0.39
*SD*	13.3	14.8	
*Female (%)*	33.3	42.3	0.31
Site of leak			
*Right chylothorax*	31	23	0.08
*Left chylothorax*	27	16	0.01*
*Bilateral chylothoraces*	7	8	1.00
*Neck*	4	18	0.004*
*Chylopericardium/Mediastinum*	3	15	0.005*
Etiology of leak			
*Lung resection*	23	25	1.0
*Esophageal resection*	18	11	0.10
*Cardiac/aortic surgery*	14	21	0.33
*Traumatic injury*	4	1	0.19
*Neck surgery*	7	16	0.07
*Other*	6	4	0.52

### Procedures

The intranodal technique is described in detail below. All procedures were performed by board- and certificate of added qualification (CAQ)-certified attending physicians with 8–20 years’ experience. Major and minor adverse events were defined according to the Society of Interventional Radiology (IR) reporting standards [6].

### Intranodal and Modified Intranodal Lymphangiography

Using real-time ultrasound guidance, bilateral inguinal lymph nodes were accessed with the outer cannula of a 25-gauge spinal needle (BD, Franklin Lakes, NJ). The needle tip was positioned in the transitional zone between the cortex and hilum of the lymph node.

Under fluoroscopic guidance, ethiodol (Lipiodol, Guerbet, LLC Princeton, NJ) was injected by hand at a rate of about 0.1 mL per 1 min via short extension tubing and a 3 mL polycarbonate syringe (Merit Medical, South Jordan, UT). If an efferent lymphatic and/or lymph node was identified, further infusion of ethiodol was performed with a balloon insufflator preloaded with 10 mL of ethiodol. The insufflator was set to administer a pressure of 3 atmospheres to propagate the contrast into the lymphatic system. A total volume of approximately 6–12 mL of ethiodol was injected into each lymph node.

Infusion of ethiodol was terminated once lymphatics at the L3 level were opacified. If the cisterna chyli or upper abdominal lymphatics were not visualized at the end of the contrast injection, the initial bolus of ethiodol was followed by injection of normal saline at 1 mL per minute to facilitate propagation of the ethiodol. Since 2016, all patients undergoing intranodal lymphangiography utilized a modified intranodal technique (mIL) in which pneumatic sequential compression devices were placed on the bilateral calves during patient positioning. The pneumatic compression devices were activated prior to sterile preparation and draping of the patient to assist with lymphatic flow as previously described [5].

### Thoracic Duct Cannulation and Embolization

Thoracic duct cannulation and embolization was performed as previously described [7]. Briefly, after the cisterna chyli and/or its contributing lymphatics were visualized with ethiodol, the lymphatic system below the cisterna chyli was accessed transabdominally under fluoroscopic guidance using a 21 or 22G Chiba needle. Using this access, a stiff 0.018″ wire (V-18, Boston Scientific, Natick, MA) was advanced into the TD followed by a 2.8F microcatheter (75 cm Rapid transit, Cordis Hialeah, FL or 100 cm Cantata, Cook Medical, Bloomington, IN). Nonionic iodinated contrast (Isovue-300) was then injected through the catheter to demonstrate the site of the chylous leak. Thoracic duct embolization was performed using a combination of platinum embolization coils (Nester, Cook Medical, Bloomington, IN) and N-butyl cyanoacrylate (N-BCA) glue (Truefill, Cordis Hialeah, FL) diluted with 1 mL of ethiodol.

### Data Collection

Electronic medical records and imaging data were reviewed for demographic data, site/etiology of the chylous leak, procedure time, fluoroscopy time, technical success, clinical success, and adverse events. Procedure time was defined as the time from preoperative time-out to completion of the procedure. Preoperative time-out was obtained from electronic medical record documentation. Completion time was obtained from the final image documented during the procedure. Technical success was defined as successful cannulation of the cisterna chyli, regardless of clinical outcome. Clinical success was defined as the resolution of the chylous leak and removal of chest tubes or drains within 2 weeks of TDE, regardless of whether the cisterna chyli was cannulated or disrupted.

### Statistical Analysis

Statistical analysis was performed using GraphPad Prism 8.4.3 software (GraphPad Software, Inc.: San Diego, CA). A two-tailed Fisher’s exact test was used to compare baseline patient characteristics between groups and differences in technical success, clinical success, and adverse event rate. An unpaired two-tailed *t* test was used to compare differences in procedural and fluoroscopic times between groups. Descriptive data are presented as mean ± standard deviation. A *p*-value of < 0.05 was considered significant.

## Results

Two hundred and thirty-nine patients underwent 263 TDEs for treatment of traumatic chylous leaks in the thorax or neck. Intranodal approach was used in 167 cases in 150 patients. Results are presented in tabular form in Table 2.

**Table 2:** Results

	IL	mIL	
*Technical success* (%)	76.5	94.2	0.002*
*Clinical success* per procedure (%)	97.2	92.3	0.27
*Clinical success* per patient (%)	85.1	83.7	0.83
*Procedure time* (min)	119.2	83.4	< 0.0001*
*Fluoroscopy time* (min)	41.7	33.8	0.02*
*Adverse event rate*	2.4	6.9	0.27

Overall, the thoracic duct was cannulated in 85.6% (143/167) of TDEs. The technical success rate was significantly higher when mIL technique was utilized compared to IL (94.2% [81/86] vs 76.5% [62/81], *p* = 0.002). Clinical success per patient was similar between the cohorts (92.3% [72/78] in mIL versus 97.2% [70/72]) in IL (*p* = 0.27). Clinical success by procedure was also similar between the cohorts (83.7% [72/86] in mIL versus 85.1% [69/81]) in IL (*p* = 0.83).

Overall successful TDEs with an IL-approach had a mean procedure time of 106.2 ± 45.3 min. Of patients with an initially unsuccessful TDE, 73.9% (17/23) underwent repeat TDE. The mean procedure time of cases performed with mIL was significantly shorter (83.4 ± 31.9 min) than the mean procedure time of cases performed with IL (119.2 ± 45.9 min) (*p* < 0.0001). Mean fluoroscopy time of cases performed with mIL (33.8 ± 17.3 min) was significantly shorter than in cases performed with IL (41.7 ± 23.2 min) (*p* = 0.02).

Adverse event rate was not significantly different between groups (6.9% [6/86] in mIL versus 2.4% [2/81] in IL) (*p* = 0.27). The minor adverse event rate was 3.6% (6/167) and included non-target embolization of glue, contained extravasation of glue, sheared wire tip, and left retroperitoneal chylous leak. The major adverse event rate was 0.6% (1/167) and included portal vein thrombosis complicated by necrotic bowel in a patient with cirrhosis. This complication is presumed to have resulted from inadvertent transgression of the portal vein or superior mesenteric and splenic vein confluence during cannulation of the lymphatics transabdominally.

## Discussion

TDE is a percutaneous, minimally invasive treatment alternative to surgical ligation in patients with post-operative chylous leaks refractory to conservative management [1]. In the past, thoracic duct opacification for identification and embolization of a lymphatic leak was achieved with the use of pedal lymphangiography (PL) [2]. PL is procedurally challenging and time-consuming as it involves cannulation of the pedal lymphatics for ethiodol administration, which must then ascend through the extremities and abdomen/pelvis prior to thoracic duct opacification and cannulation [3]. IL involves administration of ethiodol into the inguinal lymph nodes, bypassing the lower extremities and obviating the need for isolation and cannulation of the pedal lymphatics [4]. The original description of IL was reported in a small number of patients undergoing TDE for traumatic chylous leaks. The present study examines the outcomes of TDE in a larger cohort of patients with traumatic chylothorax or neck leaks using an intranodal approach overall with a comparison between two cohorts—one with the initially described IL technique and the second with a modified IL (mIL) technique incorporating sequential pneumatic compression devices to assist with lymphatic flow; and hence, ethiodol propagation toward the cisterna chyli and thoracic duct.

In the present study, procedure time, fluoroscopic time, technical outcomes, clinical outcomes, and adverse event rates for patients undergoing TDE via intranodal approach for post-surgical chylous leaks in the thorax and neck are reported. The analysis of this data demonstrates TDE using mIL is an improvement in procedural technique compared to IL. In particular, there has been a reduction in overall procedure time and improvement in technical success on a per case basis. The reduced procedure time supports the increasing ease of the IL method suggested in publications first describing the method for TDE and early descriptions of mIL which demonstrated decreased lymphatic transit time of ethiodol from inguinal nodes to the cisterna chyli. The increased technical success and decreased average procedure time demonstrate that with experience and modifications to improve lymphatic flow by use of pneumatic compression devices on the bilateral calves, TDE with mIL can be performed successfully in less than 2 h (83.4 ± 31.9 min).

A systemic review and meta-analysis including 407 patients with chylothorax from 9 studies is the largest published series on TDE and found the pooled technical and clinical success rates of TDE to be 63.1% and 79.4%, respectively [7]. This is in comparison with the present study with overall technical success rate of 85.6% (143/167) and a clinical success rate of 94.6% (142/150) across all TDEs regardless of IL method. These improved outcomes may at least in part be due to the IL and mIL approaches to TDE compared to PL. Only 11.3% of the subjects in the aforementioned meta-analysis had TDE using an IL approach, with the remaining 88.7% relying on PL to opacify the cisterna chyli. The observed differences in technical and clinical success compared to those reported in the meta-analysis may also in part be explained by inclusion of chylous leaks of any etiology, including non-traumatic leaks of malignant or congenital origins in the meta-analysis compared to the inclusion of only traumatic and post-surgical leaks in the present study.

There are several limitations to this study. First, the study is a retrospective review of patients referred to IR for embolization of chylous leaks over an extended period of time. Many of the patient referrals came from outside health systems over a long period of time; and thus, the exact duration and volume of chylous leaks in each patient is unknown over the entirety of study period. There were significantly more left-side chylous leaks in IL cohort and significantly more chylous pericardial/mediastinal and neck leaks in the mIL cohort. Additionally, as all TDEs in this series were performed by two operators at a tertiary center with a large volume of lymphatic interventions, the results and outcomes may not be reproducible in all practice settings and the effect of accumulated experience by the operators cannot be accounted for in procedural outcomes.

## Conclusion

Overall, TDE via intranodal approach for traumatic chylothorax has a high clinical success rate approaching 95%. While clinical success rate of mIL was similar to IL, technical success rate and mean procedure and fluoroscopic times were significantly improved. Findings suggest modified intranodal lymphangiography should be utilized for TDE to treat traumatic chylothorax.

## References

[1] Cope C, Kaiser LR. Management of unremitting chylothorax by percutaneous embolization and blockage of retroperitoneal lymphatic vessels in 42 patients. J Vasc Interv Radiol. 2002;13(11):1139–48. 10.1016/S1051-0443(07)61956-3. 12427814 10.1016/s1051-0443(07)61956-3

[2] Cope C. Percutaneous thoracic duct cannulation: feasibility study in swine. J Vasc Interv Radiol. 1995;6(4):559–64. 10.1016/S1051-0443(95)71134-4. 7579864 10.1016/s1051-0443(95)71134-4

[3] Pomerantz M. Lymphangiography. Surg Clin North Am. 1969;49(6):1969. 5359845

[4] Nadolski GJ, Itkin M. Feasibility of ultrasound-guided intranodal lymphangiogram for thoracic duct embolization. J Vasc Interv Radiol. 2012;23(5):613–6. 10.1016/j.jvir.2012.01.078. 22440590 10.1016/j.jvir.2012.01.078

[5] Meisinger QC, O’Brien S, Itkin M, Nadolski GJ. Use of sequential pneumatic compression devices to facilitate propagation of contrast during intranodal lymphangiography. J Vasc Interv Radiol. 2017;28(11):1544–7. 10.1016/j.jvir.2017.07.035. 28935473 10.1016/j.jvir.2017.07.035

[6] Khalilzadeh O, Baerlocher MO, Shyn PB, Connolly BL, Devane AM, Morris CS, Cohen AM, Midia M, Thornton RH, Gross K, Caplin DM, Aeron G, Misra S, Patel NH, Walker TG, Martinez-Salazar G, Silberzweig JE, Nikolic B. Proposal of a new adverse event classification by the society of interventional radiology standards of practice committee. J Vasc Interv Radiol. 2017;28(10):1432-1437.e3. 10.1016/j.jvir.2017.06.019. 28757285 10.1016/j.jvir.2017.06.019

[7] Kim PH, Tsauo J, Shin JH. Lymphatic interventions for chylothorax: a systematic review and meta-analysis. J Vasc Interv Radiol. 2018;29(2):194-202.e4. 10.1016/j.jvir.2017.10.006. 29287962 10.1016/j.jvir.2017.10.006

